# The Molecular Mechanisms Underlying Dercum’s Disease: Exploring the Intersection of Obesity, Pain, and Inflammation

**DOI:** 10.3390/ijms262211130

**Published:** 2025-11-18

**Authors:** Claudia Reytor-González, Emilia Jiménez-Flores, Melannie Toral-Noristz, Martín Campuzano-Donoso, Náthaly Mercedes Román-Galeano, Daniel Simancas-Racines

**Affiliations:** 1 Centro de Investigación en Salud Pública y Epidemiología Clínica (CISPEC), Facultad de Ciencias de la Salud Eugenio Espejo, Universidad UTE, Quito 170527, Ecuador; claudiareytor@gmail.com (C.R.-G.); mariae.jimenez@ute.edu.ec (E.J.-F.); nathalyroman0001@gmail.com (N.M.R.-G.); 2 Escuela de Medicina, Pontificia Universidad Católica del Ecuador, Santo Domingo 230203, Ecuador; 3 Escuela de Medicina, Universidad Espíritu Santo, Samborondón 0901952, Ecuador; melannietoral@uees.edu.ec; 4 Independent Researcher, Quito 170102, Ecuador; martincd01@hotmail.com; 5 Facultad de Salud y Bienestar, Pontificia Universidad Católica del Ecuador, Quito 170143, Ecuador

**Keywords:** Dercum’s disease, adiposis dolorosa, chronic pain, obesity, inflammation, therapeutic strategies

## Abstract

Obesity is increasingly recognized not only as a metabolic disorder, but also as a state of chronic low-grade inflammation that predisposes to systemic complications. Within this context, Dercum’s disease (DD), or adiposis dolorosa, emerges as a rare yet debilitating disorder characterized by painful subcutaneous lipomas, most commonly affecting middle-aged women. Despite its clinical impact, DD remains underdiagnosed and is often misclassified as lipedema, fibromyalgia, or lipomatosis, complicating prevalence estimates and hindering the development of targeted interventions. Current evidence suggests that DD represents a distinctive model of inflammatory obesity, where adipose tissue actively contributes to pain generation rather than serving as a passive fat reservoir. Histological and molecular findings point to adipose tissue dysfunction, immune cell infiltration, and elevated secretion of pro-inflammatory adipokines, signals which appear to fuel systemic low-grade inflammation, perineural immune interactions, and nociceptor sensitization. Peripheral mechanisms further shape the clinical phenotype. While familial clustering suggests possible genetic contributions, no definitive markers have been identified, and the role of obesity-induced epigenetic modifications remains unexplored. Therapeutic strategies remain largely symptomatic, including analgesics, antidepressants, physical rehabilitation, and surgical excision of lipomas, whereas molecularly targeted and diet-based interventions are still experimental. This article discusses the pathophysiology of DD, current treatments, and future perspectives, emphasizing that advancing patient registries, omics-based analyses, and interdisciplinary clinical trials will be crucial to elucidate disease mechanisms and guide novel therapies. Improved understanding of DD may not only enhance patient care, but also provide broader insights into the interplay between obesity, inflammation, and chronic pain.

## 1. Introduction

Obesity is a major global health issue, with a higher prevalence in women (40.4%) compared to men (35.0%), as reported in a cross-sectional study by Flegal et al. [1], and is linked to numerous noncommunicable diseases such as cardiovascular disease, type 2 diabetes, and various cancers [2]. Beyond its metabolic consequences, obesity is now recognized as a chronic low-grade inflammatory condition, contributing to oxidative stress and impaired immune function [3]. Excess energy intake leads to adipocyte hypertrophy and hypoxia, triggering immune cell infiltration and the activation of pro-inflammatory M1 macrophages [4]. Adipose tissue, through cytokine secretion, sustains systemic inflammation—a hallmark of metabolic syndrome—further aggravated by neutrophil elastase activity and rhythmic macrophage secretion of Tumor Necrosis Factor Alpha (TNF-α) and interleukin-6 (IL-6) [5]. This chronic inflammation promotes insulin resistance, ectopic fat deposition, vascular dysfunction, and multi-organ damage [4,6]. The rise in obesity is largely driven by increasingly obesogenic environments, highlighting the urgent need for preventive strategies and research [7].

In this context, Dercum’s disease, a condition closely associated with obesity, emerges as a significant disorder of interest. DD, or adiposis dolorosa, is a rare disorder of the connective tissue, primarily marked by chronic and often debilitating pain that manifests as a burning or searing sensation in the subcutaneous fat tissue. These painful lipomas occur predominantly in the arms, legs, and torso, without causing changes to the skin. The disease can present in different forms: a generalized, diffuse variant with smaller, less noticeable deposits that are difficult to feel, and a nodular type with larger, more prominent lipomas. Regarding the epidemiology of DD, most cases are isolated, though a few familial instances have been reported. It predominantly affects adults, especially those between the ages of 35 and 50, and is far more common in women, with female-to-male ratios ranging from 5:1 to as high as 30:1 [8]. However, accurately determining its prevalence is complicated by issues such as underdiagnosis and misclassification. These diagnostic challenges arise because the disease shares symptoms with several other conditions, such as fibromyalgia, lipoedema, and lipomatosis [8]. As a result, misdiagnoses may lead to overestimating prevalence, while underdiagnosis could result in an underestimation. Although it is listed as a disease of unknown prevalence by the National Organization of Rare Disorders [9], a narrative review by Munguia et al. [10] estimates an upper limit of 150,000 cases in the United States—below the 200,000-case threshold used to define rare diseases under the U.S. Orphan Drug Act.

Since the pathophysiology and underlying mechanisms of pain in DD remain poorly understood, with current research failing to fully elucidate the etiology of the condition, DD is particularly challenging for healthcare providers to diagnose and manage effectively [8,10,11]. As a result, there is a pressing need for further investigation into the molecular mechanisms driving DD. The aim of this review is to synthesize the current molecular evidence regarding the disease’s pathogenesis and to propose a comprehensive framework for future research and therapeutic approaches.

## 2. Clinical Characteristics and Differential Diagnosis

### 2.1. Clinical Features

Dercum’s disease, also known as adiposis dolorosa, is a rare condition primarily characterized by persistent pain in subcutaneous fat tissue. Diagnosis is clinical and based on excluding other disorders associated with lipomas [8]. The core diagnostic criteria include chronic adipose tissue pain lasting more than three months, typically affecting individuals with overweight or obesity, that is often unresponsive to standard analgesics [11,12]. Patients commonly report episodic flares of pain associated with soft, palpable, well-defined lipomas, most frequently located in the limbs and abdominal region [8,11]. Lipomas can occur in almost any region of the body, although less frequently in the head and neck, and the pain can range from mild tenderness to severe, spontaneous episodes, and is often described as aching, burning, or stabbing. It may present symmetrically or be localized to specific limbs, and, in some cases, may also involve the skeletal system [13]. Classification of DD is largely descriptive, based on the size and distribution of lipomatous deposits, which may be localized or widespread. While nodular forms are more easily identifiable, the generalized diffuse variant may involve smaller, less detectable fat deposits. Importantly, the overlying skin generally appears normal, without discoloration or ulceration. Atypical and mixed forms have also been reported, reflecting ongoing gaps in understanding the disease’s pathophysiology [14,15,16].

The condition most commonly affects obese women, particularly during or after menopause, with women being diagnosed 5 to 30 times more often than men [12]. DD is closely associated with excess body fat, as the abnormal growths consist of adipose tissue, and is frequently accompanied by several systemic and neurological symptoms [17]. In fact, a study by Herbst et al. [18], based on a survey of 110 patients diagnosed with DD, revealed a range of frequently reported symptoms. These included chronic pain, fatty growths resistant to weight loss, easy bruising, sleep disturbances (both poor sleep quality and clinical insomnia), memory problems, depression, concentration difficulties, anxiety, palpitations, breathing issues, diabetes, gastrointestinal disturbances like bloating and constipation, fatigue, muscle and joint pain, and generalized weakness, but mood instability, insomnia, and even cognitive impairment or dementia have also been reported [8,11]. Additionally, reduced sleep quality in obese individuals may exacerbate the fatigue and weakness commonly experienced in DD [19], and a higher body mass index has also been linked to increased prevalence of anxiety and certain personality disorders [20].

Numerous case reports have documented the strong link between Dercum’s disease and obesity [11,12,14,18,21,22,23,24,25,26]. On a cellular level, obesity causes profound alterations in fat tissue composition—such as enlarged adipocytes, infiltration by immune cells, and changes in the extracellular matrix (ECM)—all of which contribute to metabolic disruption [27]. As adipose tissue enlarges, insufficient blood vessel growth can result in reduced capillary density and localized hypoxia. Hypoxia is considered one of the earliest pathophysiological events in obese fat tissue, playing a key role in fibrosis by activating hypoxia-inducible factor 1 (HIF1α) and also promoting inflammation and lipid abnormalities [27]. Additionally, high triglyceride levels have been linked to the formation of lipomas [28].

More recently, attention has turned to hormonal factors, particularly estrogen, in adipose tissue biology and related disorders. Estrogen influences a wide range of metabolic processes, including fat cell development, insulin sensitivity, secretion of adipokines, breakdown and storage of fats, and immune regulation [29]. Estrogen receptors, especially estrogen receptor alpha (Erα), are also involved in modulating immune and fibrotic responses in adipose tissue [30]. Moreover, estrogen suppresses pro-inflammatory activity, and women undergoing surgical menopause often exhibit elevated markers of inflammation [31]. During menopause, falling estradiol levels and a relative increase in estrone—produced primarily in fat tissue—alter both fat distribution and metabolic function [32]. This decline in estrogen signaling may also enhance adipose inflammation and nociceptive sensitivity, helping to explain the higher prevalence of Dercum’s disease in postmenopausal women [33]. Estrogen deficiency can, thus, contribute to metabolically harmful fat expansion via adipocyte hypertrophy [34,35].

An illustrative example is lipedema, which has been proposed as an estrogen-sensitive adipose disorder possibly initiated by caveolin-1 (CAV1) dysfunction [33]. CAV1 is a membrane-bound protein that helps organize signaling molecules within caveolae and regulates pathways involving estrogen receptors [36,37]. When CAV1 function is disrupted, estrogen signaling may be impaired, potentially resulting in abnormal transcription and promoting tumor-like processes [31]. Estrogen also governs tissue-specific immune responses, and deficiencies or receptor abnormalities have been linked to metabolic syndrome and obesity [29]. This connection between estrogen signaling and CAV1 may contribute to the abnormal fat accumulation observed in certain adipose disorders, highlighting its significance in the regulation of fat tissue and related metabolic or inflammatory conditions.

### 2.2. Clinical Subtypes

A limited understanding of the disease’s causes and progression hinders the development of a pathophysiological classification [8]. However, there is a classification where four subtypes of DD are proposed (Table 1).

However, these subtypes only describe the size or location of the nodules and do not provide additional information about the condition, such as family history, previous medical history, or the presence of angiolipomas [10,38,39]. Therefore, another proposed classification is based on etiology, and it includes obesity, trauma, healing disorder, familial multiple lipomatosis (FML), and angiolipoma. For example, the healing disorder subtype of DD may be due to sequelae from infections such as Lyme disease, the FML subtype is, in part, genetically determined, and the obesity subtype is further explained below [10].

### 2.3. Differential Diagnosis

Diagnosing DD presents a significant clinical challenge due to overlapping features with various adipose tissue disorders and systemic syndromes [24]. A comprehensive evaluation of potential differential diagnoses is essential in this setting. One of the most frequently mistaken conditions is fibromyalgia, characterized by widespread muscle pain, fatigue, and cognitive difficulties. However, unlike DD, fibromyalgia does not involve lipomas, and the pain originates in muscle and connective tissue rather than fat. Additionally, DD tends to produce more intense and widespread pain [26]. Another condition to consider is lipedema, which predominantly affects women and leads to symmetrical fat buildup in the lower extremities. Although the affected fat is sensitive and prone to bruising, it does not form distinct painful nodules. Lipedema typically spares the feet and is not associated with systemic features such as fatigue or cognitive issues [24]. Research further indicates that individuals with DD frequently experience other chronic pain syndromes, including fibromyalgia, abdominal pain, and migraines, and are more likely to report symptoms such as shortness of breath. Conversely, people with lipedema often exhibit fibrotic tissue, venous insufficiency, foot swelling and joint hypermobility [40]. Lymphedema, by contrast, presents with asymmetrical swelling resulting from impaired lymphatic drainage, often accompanied by skin changes. Unlike DD, it does not include painful lipomas, and any pain tends to be minimal [24]. FML is another inherited disorder, transmitted in an autosomal dominant pattern, that results in multiple slow-growing lipomas, typically located on the forearms and thighs. These growths are generally painless, and systemic symptoms are absent, which helps distinguish FML from DD [8,24]. Moreover, the lipomas in FML are encapsulated and usually do not cause discomfort [39]. Subcutaneous angiolipomas, a vascularized form of lipoma, differ from classic lipomas by being painful to touch. On ultrasound, they appear as well-defined hyperechoic nodules, and Doppler imaging may reveal internal blood flow, which helps differentiate them from simple lipomas [26].

Beyond these conditions, various syndromic and hormonal disorders can exhibit features similar to DD. For instance, Madelung’s syndrome (also known as benign symmetric lipomatosis) presents with symmetrical, poorly defined fat deposits, mainly around the neck, trunk, and proximal limbs. This condition primarily affects non-obese, middle-aged men with a history of alcohol use and is often associated with neurological manifestations such as peripheral neuropathy, features that are uncommon in DD, which predominantly affects obese women [26,39]. Cushing’s syndrome also mimics DD in terms of general symptoms such as obesity, fatigue, and psychiatric disturbances. However, the associated pain in Cushing’s syndrome is not linked to nodular fat. Therefore, it is important for clinicians to consider and exclude Cushing’s when evaluating patients with subcutaneous lipomas [8,26].

Several genetic disorders may include lipomas as part of their clinical spectrum, yet they remain distinct from DD due to additional systemic signs. These include Proteus syndrome, PTEN hamartoma syndrome, Gardner syndrome, Cowden’s disease, and Bannayan–Riley–Ruvalcaba syndrome, which often involve macrocephaly, multiple hamartomas, or an elevated cancer risk, none of which are typical in DD [39]. Other rare disorders such as Weber–Christian disease, erythema nodosum, erythema induratum, neurofibromatosis type 1, Fröhlich syndrome, lipodystrophia progressiva, and congenital lipomatosis are also listed in the differential diagnosis for DD [26,39]. Additionally, liposarcomas should be considered. These malignant tumors tend to be solitary, may grow quickly, and exhibit features suggesting malignancy, contrasting sharply with the multiple benign lipomas characteristic of DD [24]. Cutaneous metastases from internal malignancies may also manifest as subcutaneous nodules, though they are usually painless and appear hypoechoic on ultrasound, unlike the typically hyperechoic benign lipomas found in DD [26].

## 3. Proposed Pathophysiology of Dercum’s Disease

The mechanisms underlying DD remain unclear, with no definitive cause established. Multiple systems are likely involved, including autoimmune processes that may explain chronic inflammation and systemic manifestations [41], endocrine disturbances such as thyroid and pituitary dysfunction, and hormonal influences, including steroid therapy [33].

Metabolic abnormalities have also been reported, including impaired glucose-to-triglyceride conversion in painful fat, altered lipid metabolism, and resistance to insulin and norepinephrine [12,33]. Additional proposed triggers include infections, chronic inflammatory conditions, alcohol use, injury, and rapid weight changes, such as after bariatric surgery [33]. Overall, the primary pathophysiological mechanisms are thought to involve adipose tissue dysfunction, systemic low-grade inflammation, and neuroinflammation with nociceptor activation [42].

### 3.1. Adipose Tissue Dysfunction

Adipose tissue dysfunction is central to metabolic disturbances in obesity and may contribute to rare conditions like DD [41,43]. In nutrient excess, fat expands via hypertrophy and hyperplasia, with hypertrophy linked to impaired glucose tolerance, elevated lipids, and systemic inflammation, whereas hyperplasia is metabolically more favorable [27,44].

Metabolically healthy individuals tend to have smaller adipocytes, while those with metabolic impairments exhibit enlarged cells, reflecting either direct contribution to disease or limited adipose expansion, leading to lipid spillover [27,45]. Exceeding fat storage capacity fosters chronic inflammation and insulin resistance, exacerbated by M1 macrophage infiltration, elevated pro-inflammatory receptors (Toll-Like Receptors, TNF receptors, and IL-1R), and activation of Nuclear Factor Kappa-light-chain-enhancer of Activated B Cells (NF-κB), which promotes inflammatory mediator production. Insulin resistance further amplifies inflammation by diminishing insulin’s anti-inflammatory and vasodilatory effects [4,46,47].

On a cellular level, excessive lipid accumulation contributes to mitochondrial dysfunction and endoplasmic reticulum stress in adipocytes. These alterations lead to unfavorable gene expression and a shift in adipokine secretion, which, in turn, exacerbates systemic insulin resistance and metabolic imbalance [29]. Estrogen signaling, particularly through ERα, has also been identified as crucial in regulating adipocyte function. Estrogens limit fat cell growth by inhibiting specific molecular pathways, and their effects differ between sexes. Female adipose tissue, especially subcutaneous fat, is more responsive to estrogen and tends to expand more in response to overnutrition [48]. In obesity, however, both sexes exhibit reduced expression of estrogen receptors in adipose tissue, promoting hypertrophy over hyperplasia and contributing to dysfunctional fat expansion [29].

**Figure 1:**
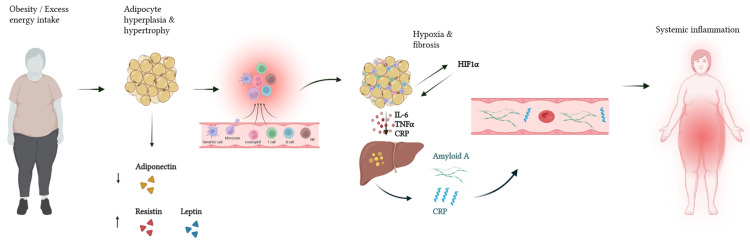
Obesity-driven adipose inflammation and metabolic dysfunction. Schematic representation of the transition from healthy to inflamed adipose tissue under conditions of chronic overnutrition. The figure illustrates macrophage infiltration, altered adipokine secretion, and hypoxia-induced fibrosis contributing to systemic inflammation and metabolic dysfunction. Key immune and molecular mediators involved in this process are shown [27,52,53,54]. Abbreviations: TNFα: tumor necrosis factor alpha; CRP: C-reactive protein; HIF1α: hypoxia-inducible factor 1-alpha.

A critical yet often overlooked feature of adipose tissue dysfunction is its inflammatory transformation in obesity (Figure 1), and in Dercum’s disease, this is marked by distinct metabolic irregularities, as painful fat deposits contain elevated levels of long-chain monounsaturated fatty acids absent in unaffected tissue, suggesting abnormal lipid processing [42]. Affected fat in DD exhibits reduced glucose-to-triglyceride conversion, reflecting disrupted carbohydrate and lipid metabolism. Lymphatic dysfunction has also been proposed as a contributing factor [49]. Near-infrared fluorescence lymphatic imaging reveals abnormal, fibrotic, and dilated lymphatic vessels within DD adipose tissue, suggesting impaired drainage and pathological interactions between lymphatic and adipose systems [33]. Such lymphatic insufficiency may underlie lipoma formation and chronic pain [49], echoing early observations by Dr. Dercum linking the lymphatic system to adipose pathology [33,41].

Histological studies of DD reveal biopsies of painful fat with increased connective tissue, resembling conventional lipomas [50,51]. Although data remain limited, findings suggest reduced metabolic activity, likely due to fibrosis and tissue remodeling. Inflammatory features, including multinucleated giant cells from activated pro-inflammatory macrophages, may contribute to abnormal fat accumulation and resistance to weight loss. In a study by Herbst et al. [51], adipose tissue biopsies were obtained from painful areas in five DD patients and five control subjects. The analysis revealed elevated IL-6 levels in DD patients, while no significant differences were found for TNF-α, IL-1β, IL-8, or IL-13. Notably, multinucleated giant cells were identified in three of the five DD patients, but in none of the control group, despite similar overall macrophage counts. Hansson et al. [50] conducted a larger study involving 53 DD patients and 52 controls, including both obese (n = 41) and non-obese individuals (n = 11). Fat biopsies from painful regions showed that the inflammatory profile in DD patients was comparable to that of obese controls but more pronounced than in non-obese controls, suggesting that the inflammatory component observed in DD may be more closely associated with obesity rather than being specific to the disease itself [33].

### 3.2. Systemic Low-Grade Inflammation

Obesity is a low-grade inflammatory condition that increases the risk of various chronic diseases [55]. It involves adipose tissue expansion, leading to hypoxia and activation of pro-inflammatory M1 macrophages [4]. This expansion drives chronic inflammation through immune cell infiltration and overproduction of cytokines such as IL-1β, IL-6, TNF-α, and Monocyte Chemoattractant Protein-1 (MCP-1). Dysfunctional adipose tissue also promotes macrophage polarization and the formation of crown-like structures around dead adipocytes, along with increased neutrophils, CD8+ cytotoxic T cells, and NK cells and a reduction in CD4+ T helper cells [3].

Although the exact cause of DD remains unclear, it is thought to involve inflammatory processes [56]. Laboratory markers like C-reactive protein (CRP) and erythrocyte sedimentation rate (ESR) are typically normal [18,39,57,58,59], but some studies have shown elevated levels of these markers in DD patients, such as a study described in a review by Hansson et al. [19], where it was found that 66% of 112 women with DD had an ESR greater than 15 mm/h, while Herbst et al. [18] reported in their study that 33.4% of DD patients had elevated CRP levels and 37.5% had increased ESR levels. Inflammation within adipose tissue has also been noted, with the presence of leukocytes and plasma cells in certain cases. Additionally, Herbst et al. [51] observed the presence of multi-nucleated giant cells in three out of five DD patients, but none of the controls (n = 5), a feature typically associated with macrophage activation [50,51]. Pathological examination of fat biopsies from DD patients also revealed an increase in connective tissue, fibrolipomas with numerous embryonic vessels, small angiomas, and capillary microthrombi, all of which may be linked to the chronic inflammatory state [3,50].

A multiplex immunoassay analyzing 37 cytokines in lipedema serum samples identified 22 cytokines present in the condition, with significantly elevated levels of IL-11, IL-28A, and IL-29. IL-29 has been associated with inflammation linked to obesity and insulin resistance, stimulating the expression of IL-8, IL-1β, and MCP-1. Both IL-28A and IL-29 are secreted by macrophages, and IL-11 promotes the proliferation of adipose-derived stem cells while inhibiting adipogenesis, contributing to the hyperplasia seen in lipedema [60].

Inflammatory processes in obesity and DD are shaped by altered adipokine secretion. Leptin promotes inflammation by increasing TNF-α and IL-6, reducing anti-inflammatory adiponectin, driving macrophage polarization toward M1, enhancing Th17 responses, and inhibiting regulatory T-cells [3]. Reduced adiponectin contributes to cardiovascular risk. Other adipokines, such as omentin-1, also have anti-inflammatory roles but are decreased in obesity, while resistin, secreted by macrophages, activates NF-κB, causing endothelial dysfunction and chronic inflammation [29,61,62]. Visfatin, produced by adipocytes and macrophages, is associated with visceral fat accumulation, elevated cardiometabolic risk, and increased mortality [29,63,64].

Subcutaneous adipose tissue (SAT) hosts immune cells, including monocytes/macrophages, mast cells, and lymphocytes, which contribute to hormonal regulation and innate immunity [33]. Mast cells, key players in inflammation and fibrosis, are implicated in DD, particularly in the angiolipoma subtype, through mast cell activation syndrome [65]. They secrete histamine, serotonin, and heparin, increasing microvascular permeability and leukocyte infiltration [66,67]. Elevated peripheral serotonin, linked to obesity, affects insulin resistance, and inhibiting its synthesis in mast cells can prevent these effects, suggesting a potential mechanism in DD [68,69]. Basophils, closely related to mast cells, are significantly elevated in DD versus lean and obese controls, promoting T Helper 2 cell (Th2) responses and further inflammation [65,66].

NK cell numbers are reduced in DD, potentially impairing adipose tissue remodeling and contributing to chronic inflammation and insulin resistance. Alongside basophils, which initiate Th2 responses, and mast cell activation, these immune alterations—absent in controls—may underlie DD’s distinctive symptoms, including painful lipomas [66,70]. Macrophages in DD predominantly adopt M1-like pro-inflammatory phenotypes typical of chronic inflammation, with evidence also suggesting the presence of M2c macrophages due to Transforming Growth Factor Beta signaling [46,47].

### 3.3. Neuroinflammation and Nociceptor Activation

Since Hansson et al. [71] suggested that sensory nerve avulsion via liposuction may relieve pain by disrupting abnormal connections between the peripheral and sensory nervous systems, pain may be mediated by perineural neuroinflammation and nociceptor activation [72]. Neuroimmune interactions are key contributors, as sensory neurons release neuropeptides such as Substance P and calcitonin gene-related peptide, which promote vasodilation, increase vascular permeability, and recruit immune cells [73]. These changes establish a pro-inflammatory microenvironment that sensitizes nociceptors. Substance P, in particular, can induce mast cell degranulation and the release of histamine and pro-inflammatory cytokines like TNF-α and IL-1β, enhancing nociceptive signaling [74]. The following two main hypotheses have emerged to explain the mechanisms of pain induction: local mechanisms involve direct peripheral inflammation and nociceptor sensitization, whereas systemic mechanisms involve persistent peripheral input leading to central sensitization and neuroinflammation in the spinal cord or brain. Both pathways may coexist, contributing to chronic pain [75].

## 4. Molecular Mechanisms of Pain

The pain commonly observed in DD cannot be solely attributed to fat accumulation. Alternative explanations have been suggested, such as compression of nerves by lipomatous tissue, alterations in nerve structure, and microvascular thrombosis [12,66].

### 4.1. Peripheral Sensitization

Peripheral sensitization refers to the process by which nociceptors become increasingly reactive to stimuli due to biochemical changes in their environment. This phenomenon, commonly observed in chronic pain conditions, is especially pronounced in inflamed adipose tissue, where an abundance of pro-inflammatory mediators modulate ion channel activity, lowering the activation threshold of nociceptors and increasing their firing probability [76].

Once considered a passive fat reservoir, adipose tissue is now understood to function as an active immunometabolic organ. It not only contains adipocytes, but also fibroblasts, endothelial cells, and immune cells such as macrophages and dendritic cells. Under inflammatory conditions, these components release a variety of signaling molecules—including cytokines, neuropeptides, reactive oxygen species, and growth factors—that sensitize nociceptors directly or indirectly by altering their ionic conductance [13].

Voltage-gated sodium channels (Navs) play a fundamental role in action potential initiation and propagation in sensory neurons. Subtypes Nav1.7 and Nav1.8, in particular, are strongly implicated in inflammatory pain states due to their enhanced expression and activity during inflammation. Pro-inflammatory agents such as TNF-α and bradykinin have been shown to modulate their function, promoting hyperexcitability of nociceptors [77]. These channels are critical therapeutic targets; pharmacological agents—including local anesthetics like lidocaine—can block Navs, leading to reduced sensory input and analgesia. Nav1.9 also contributes to this process, although its role is less prominent compared to Nav1.7 and Nav1.8 [77,78,79].

Another major group of ion channels involved in nociceptor sensitization is the ac-id-sensing ion channels (ASICs), which detect changes in extracellular pH. These voltage-independent, proton-gated channels are particularly responsive to acidic environments—common under various physiological and pathological conditions, such as neuronal activation, inflammation, and reduced blood flow due to microvascular obstructions like microthrombi—and contribute to pain signaling by allowing sodium influx into neurons [80]. As an example, joint inflammation is usually associated with a decrease in local synovial fluid pH to as low as pH 6, within the physiological pH range for activation of ASIC3 [80,81]. ASIC1 and ASIC3 are the most relevant subtypes in pain physiology: the former is primarily associated with primary hyperalgesia, while the latter mediates secondary hyperalgesia, especially in musculoskeletal and inflammatory pain models [82].

Tissue acidosis not only activates these channels, but also shapes the local immune response. ASICs are expressed in both neuronal and non-neuronal cells—including immune cells—where their activation enhances the expression of maturation markers (e.g., CD80, CD86, and Major Histocompatibility Complex Class II) and promotes the release of cytokines such as IL-1β [80,83]. Furthermore, inflammatory mediators like Nerve Growth Factor (NGF), ILs, and serotonin upregulate ASIC expression in dorsal root ganglia neurons, amplifying pain responses by increasing the intensity and frequency of ASIC-mediated currents. Thus, ASICs are central in linking metabolic changes in inflamed tissue to nociceptor activation and immune modulation [84]. Transient receptor potential vanilloid 1 (TRPV1), another key ion channel in peripheral sensitization, is a non-selective cation channel activated by a wide range of stimuli, including low pH and endogenous metabolites [85,86,87]. It is highly expressed in sensory neurons and is co-localized with markers like substance P and Calcitonin Gene-Related Peptide. TRPV1 activation leads to an influx of calcium ions, triggering signaling cascades via phospholipase C, which catalyzes the hydrolysis of Phosphatidylinositol 4,5-bisphosphate into Inositol 1,4,5-trisphosphate and diacylglycerol [88]. These pathways further sensitize the membrane, increasing neuronal excitability and nociception [89].

Moreover, TRPV1 is intricately involved in neurogenic inflammation, a process characterized by vasodilation, edema, and immune cell recruitment following nociceptor overactivation. This mechanism is relevant to various inflammatory diseases, including those involving widespread or systemic symptoms such as Dercum’s disease [13,89]. Research also suggests that chronic inflammation modulates TRPV1 expression and function, making it a promising target in the treatment of persistent inflammatory pain [88].

### 4.2. Pain Mediators

Pain resulting from inflammation or tissue injury is mediated by a complex network of biochemical signals that interact with sensory neurons to initiate and sustain nociceptive transmission [90]. Among the most studied and clinically relevant of these mediators are prostaglandins, bradykinin, and pro-inflammatory cytokines, all of which contribute to the development of acute and chronic pain through sensitization of nociceptors and modulation of neuronal excitability [91,92,93,94].

Prostaglandins, particularly prostaglandin E2 (PGE2), are lipid-derived signaling molecules produced via the cyclooxygenase (COX) pathway from arachidonic acid. Unlike many other eicosanoids, PGE2 is synthesized de novo following cellular activation and is rapidly released into the extracellular space. This mediator is involved in vasodilation and modulation of immune responses and plays a dual role by exhibiting both pro- and anti-inflammatory properties depending on the context [95]. In the setting of inflammation, however, PGE2 plays a pivotal role in eliciting pain, since it sensitizes peripheral sensory neurons. Its action is mediated through G protein-coupled receptors, which activate intracellular signaling cascades such as the cyclic adenosine monophosphate/protein kinase A (PKA) pathway, leading to phosphorylation and sensitization of TRPV1 channels and voltage-gated sodium channels. This cascade ultimately reduces nociceptor activation thresholds and amplifies pain perception [96].

Importantly, bradykinin also contributes significantly to peripheral sensitization. It is a vasoactive peptide that promotes vasodilation, increases vascular permeability, and directly activates nociceptors. Bradykinin exerts its effects through B1 and B2 receptors, with most physiological responses—such as pain and PGE2 release—being mediated by B2 receptor activation [97]. Bradykinin stimulation leads to activation of extracellular signal-regulated kinases 1 and 2 and p38 mitogen-activated protein kinase (MAPK) signaling pathways, which then trigger NF-κB-mediated upregulation of COX-2 expression, thereby enhancing PGE2 synthesis and perpetuating the inflammatory pain cycle [93].

In the inflamed microenvironment, nociceptors are also influenced by a wide range of cytokines, such as tumor necrosis factor-alpha (TNF-α), IL-1β, and IL-6. These signaling proteins are secreted by immune cells—including neutrophils, macrophages, and glial cells—and contribute to both the initiation and maintenance of pain [91]. Macrophages, particularly the M1 phenotype, release abundant quantities of these cytokines along with chemokines, prostaglandins, and NGF, all of which modulate nociceptor sensitivity and amplify inflammatory signaling [91,98]. Upon interaction with their respective receptors on nociceptive neurons, these cytokines activate intracellular kinases such as PKA, Protein Kinase C, and p38 MAPK, resulting in increased activity of ion channels like TRPV1 and Nav1.8 and further enhancing pain responses [91,99,100,101].

Neuroinflammation also contributes significantly to chronic pain. Following tissue injury, immune and glial cells are recruited to the periphery and central nervous system, where they release pro-inflammatory mediators that communicate bidirectionally with nociceptors [102]. These interactions promote peripheral sensitization, characterized by reduced activation thresholds and exaggerated responses to stimuli. The release of inflammatory and chemical mediators such as bradykinin, prostaglandins, K^+^, and H^+^ into the extracellular space leads to a cascade of events, including mast cell degranulation, histamine release, and upregulation of pain-sensitizing molecules like NGF and substance P [103,104,105]. This ultimately results in the sensitization of surrounding nerve endings and the manifestation of symptoms such as hyperalgesia and allodynia [106].

Moreover, there is evidence that the excitatory actions of inflammatory mediators can be potentiated by environmental conditions such as tissue acidosis. For example, the co-presence of low extracellular pH can synergistically enhance nociceptor activation by bradykinin or PGE2, suggesting that inflammatory pain is the result of both biochemical and microenvironmental changes [107]. This multifaceted response is further intensified by immune-mediated structural changes, such as nerve remodeling, which can lead to persistent hypersensitivity even after resolution of the initial inflammation [96].

Importantly, these inflammatory pain mechanisms share significant overlap with those observed in nociplastic pain conditions, such as fibromyalgia. Nociplastic pain is defined by altered nociceptive processing within the central nervous system, leading to hypersensitivity in the absence of clear peripheral tissue damage. This concept—formerly referred to as “central sensitization”—involves dysregulated ascending and descending pain pathways, impaired inhibitory control, and amplification of pain signals [108]. Pro-totypical nociplastic syndromes like fibromyalgia, chronic migraine, and irritable bowel syndrome frequently present with coexisting symptoms such as fatigue, cognitive impairment, mood disturbances, and sensory hypersensitivity [108,109].

Several molecular patterns are shared between inflammatory and nociplastic pain. Elevated levels of IL-6, IL-8, TNF-α, and IL-1β—all classic mediators in inflammation—have also been identified in fibromyalgia, suggesting the presence of low-grade systemic inflammation [110]. These cytokines contribute to central sensitization by promoting glial cell activation and enhancing nociceptive transmission within the spinal cord and brain. Moreover, substances like NGF and PGE2, implicated in inflammatory pain, are also involved in maintaining central hyperexcitability in fibromyalgia [91]. Shared involvement of ion channels such as TRPV1 and Nav1.8, as well as intracellular cascades like MAPK and NF-κB, further supports the mechanistic overlap between the two pain types.

## 5. Genetic and Epigenetic Considerations

Expanding on previous descriptions of Dercum’s disease and its clinical features, recent studies increasingly point to the potential involvement of genetic and epigenetic mechanisms in its etiology. Although the precise pathogenesis remains unclear, growing evidence indicates that both genetic predisposition and obesity-associated epigenetic alterations may play a substantial role in its development. While most cases of Dercum’s disease appear to be sporadic, multiple publications have reported familial occurrences, often displaying a pattern consistent with autosomal dominant inheritance and variable expression [12,22]. Campen et al. [111] described familial clustering, and reported a male patient with a family history showing features overlapping with both DD and familial multiple lipomatosis. Such findings strengthen the hypothesis that a proportion of cases may have a hereditary basis. Li et al. documented a family with multiple members exhibiting lipomatous features, supporting a potential genetic origin [24]. Similarly, familial aggregation has been observed in studies on FML, a condition characterized by painless lipomas, which some researchers consider distinct yet related. Mejia Granados et al. [112] reported that FML follows an autosomal dominant inheritance pattern, further suggesting genetic continuity with DD.

Despite compelling evidence suggesting heritability, efforts to identify a definitive genetic cause for Dercum’s disease have, thus far, been inconclusive. Molecular studies of lipomas have identified chromosomal abnormalities, particularly translocations affecting chromosome 12q15 and the High-Mobility Group AT-Hook 2 (HMGA2) gene, a known regulator of adipocyte differentiation and proliferation [111,112]. Although these alterations have not been directly linked to DD, the overlap with FML and similar lipomatous pathologies implies that shared genetic mechanisms may be involved. Furthermore, Rasmussen et al. [49] observed a unique lymphovascular phenotype in patients with Dercum’s disease, including sluggish lymphatic flow and structural lymphatic remodeling, which may reflect underlying genetic defects in vascular or lymphatic development. In some cases, lipomas displayed characteristics akin to tertiary lymphoid structures, suggesting an immunogenetic component in disease progression.

Additional support for this theory comes from recent immunophenotyping studies, which have identified a distinct inflammatory profile in DD. Dupuis et al. [41] found elevated leukocyte and platelet counts, increased basophils, and reduced natural killer (NK) cells in affected patients compared to both obese and lean controls. Given that basophils and mast cells contribute to Th2 immune responses and serotonin release—a mediator implicated in both obesity and pain—these immune alterations are noteworthy. A reduction in NK cells may impair the remodeling of adipose tissue, leading to sustained inflammation and insulin resistance [41,105]. Although these findings are not strictly genetic, they underscore the possibility that immune dysregulation may be influenced by genetic vulnerability.

Obesity is not merely characterized by excess fat but is now understood as a chronic, low-grade inflammatory condition with systemic metabolic and immunological effects. In Dercum’s disease, obesity may serve as both a trigger and amplifier by inducing epigenetic modifications in adipose tissue. These epigenetic changes—such as DNA methylation, histone modifications, chromatin remodeling, and regulation via non-coding RNAs—are heritable yet reversible alterations in gene expression that do not modify the underlying DNA sequence.

Ziadlou et al. [113] emphasized the immunological activity of SAT, which undergoes significant remodeling during obesity. Persistent low-grade inflammation in SAT drives stable epigenetic reprogramming of resident immune and stromal cells, promoting pro-inflammatory states. This so-called “epigenetic memory” may help sustain inflammation even after weight loss and could contribute to the chronic pain and adipose dysfunction seen in DD. Methylation shifts in genes involved in cytokine regulation and immune checkpoints can further exacerbate immune dysregulation, linking local adipose tissue changes to systemic outcomes [114,115].

In support of this view, Long et al. [116] investigated the systemic epigenetic effects of obesity, revealing DNA methylation changes in anti-inflammatory genes and hypomethylation in pro-inflammatory cytokine genes. These alterations promote NF-κB activation and skew immune polarization toward inflammatory macrophages and T cells. Moreover, dysregulated microRNAs worsen adipose tissue inflammation by blocking M2 macrophage polarization and stimulating fibrotic signaling. In Dercum’s disease, the presence of persistently inflamed, painful lipomas may reflect these epigenetically reprogrammed adipose environments. The altered profile of cytokines and adipokines, immune cell infiltration, and disrupted adipocyte turnover all mirror features of obesity-induced adiposopathy.

Histone deacetylases (HDACs) and sirtuins (e.g., SIRT1) are also key epigenetic regulators that influence chromatin structure and gene transcription related to immune response and energy metabolism. In obesity, altered HDAC and SIRT1 activity may shift gene expression patterns in adipocytes and immune cells toward a sustained pro-inflammatory state. These changes can persist over time, contributing to chronic inflammation and metabolic dysfunction, and may be relevant to the persistent symptoms observed in DD [117].

Environmental exposures further shape the epigenetic landscape. Ghosh et al. [118] summarized two decades of findings linking early-life exposures, nutrition, stress, pollution, and physical inactivity to long-term epigenetic programming that increases vulnerability to obesity and metabolic disease. These factors affect gene expression through methylation and non-coding RNA activity, influencing immune tolerance, metabolism, and neuroendocrine regulation. Given that many patients with Dercum’s disease experience obesity and neuropsychiatric symptoms, these environmentally driven epigenetic mechanisms are likely contributors to disease onset and progression.

Multiple modifiable environmental variables may influence epigenetic regulation and adipose health in DD. Christofides and Gonzalez-Campoy highlighted various “adipocyte disruptors” that contribute to adiposopathy—a term for dysfunctional adipose tissue. Among these, circadian rhythm disruption plays a critical role. Altered sleep–wake cycles—often caused by artificial light exposure or poor sleep hygiene—can impair hypothalamic–pituitary–adrenal (HPA) axis function, disturb ghrelin/leptin regulation, and reduce GLP-1 secretion, thereby promoting visceral fat accumulation and metabolic imbalance. These changes exacerbate inflammation and pain, both hallmark features of DD [119].

Vitamin deficiencies, especially in vitamins A and D, have also been implicated. Vitamin A influences Retinol Binding Protein 4 and glucose metabolism, while vitamin D has known roles in suppressing inflammatory macrophages. Deficiencies in these nutrients can intensify pro-inflammatory signaling within adipose tissue and contribute to dysfunction. Additionally, the gut microbiome acts as a critical interface between environmental stimuli and metabolic regulation. Dysbiosis—a disruption of microbial balance—can affect serotonin metabolism, fatty acid absorption, and immune homeostasis. Alterations in the gut–brain axis and interventions such as fecal microbiota transplantation have been shown to modulate adipocyte behavior and systemic inflammation, offering new insights into the role of the microbiota in adipose-related diseases [119].

In sum, Dercum’s disease serves as a prime example of how genetic susceptibility and environmentally driven epigenetic programming can intersect in the pathophysiology of chronic inflammatory conditions. Although most cases are sporadic, familial clustering and inheritance patterns consistent with autosomal dominance suggest a hereditary component, potentially overlapping with genes implicated in familial multiple lipomatosis. Genetic anomalies such as HMGA2-related chromosomal rearrangements, along with immune dysregulation and lymphatic abnormalities, reinforce this genetic link. At the same time, obesity-induced epigenetic alterations provide a compelling model to explain the persistent inflammation, immune activation, and pain observed in this disease. Epigenetic disruptors—including circadian misalignment, poor nutrition, and microbial imbalance—may further aggravate this adipopathic state. Deeper understanding of these mechanisms paves the way for targeted interventions, including lifestyle modifications and epigenetic therapies, to improve patient outcomes in Dercum’s disease.

## 6. Therapeutic Approaches and Future Perspectives

The management of DD is tailored to each patient, with an emphasis on alleviating pain rather than achieving a cure. A multidisciplinary approach is typically advised. While analgesics are the primary medications used, many patients show limited response to non-steroidal anti-inflammatory drugs (NSAIDs) [42].

### 6.1. Current Symptom-Based Treatments

Despite the presence of the various potential pain mechanisms outlined earlier, there is currently no definitive cure for DD. As such, the focus of treatment is on alleviating pain through a variety of symptomatic therapies, each tailored to the individual’s response [11].

The initial pharmacologic approach typically includes NSAIDs, although their effectiveness in pain relief is often limited. Traditional painkillers, such as opioids, show varying success, with certain buprenorphine formulations proving particularly effective for severe pain cases [120,121]. In a large-scale survey, narcotics were linked to a 97.3% improvement in pain, followed by antidepressants (88.9%), NSAIDs (88.8%), lidocaine (87.5%), hot baths (81.4%), lipoma removal (78.5%), corticosteroids (68.4%), heat (64.8%), cold therapy (19.1%), and physical therapy (18.9%) [18].

Lidocaine, administered topically or intravenously, alleviates DD pain by blocking peripheral sodium channels, including tetrodotoxin-resistant types, targeting neuropathic mechanisms [42,72]. Its effects may also involve modulation of the sympathetic nervous system, though the mechanisms are not fully understood [72]. Intravenous ketamine, an N-Methyl-D-Aspartate receptor inhibitor, has also been used to reduce pain and opioid dependence [42,72,122].

Corticosteroids, such as prednisone and intralesional methylprednisolone, yield mixed results, with some patients experiencing relief while others worsen, and high doses potentially aggravate disease progression [42]. Tricyclic antidepressants, particularly amitriptyline, alleviate pain and stabilize mood via norepinephrine reuptake inhibition. Pregabalin and gabapentin modulate neuronal excitability and neurotransmitter release; pregabalin improves pain and sleep by reducing substance P and glutamate, whereas gabapentin may exacerbate edema in some individuals [40,42].

Complementary treatments have gained interest in DD management. Deoxycholic acid, used for cosmetic fat reduction, shows potential for lipoma management but carries risks such as vascular complications, alopecia, and skin lesions [123,124]. Manual lymphatic drainage and subcutaneous massage targeting fat and fascia can reduce pain and lipoma size [8,72,125,126]. Frequency Rhythmic Electrical Modulation System therapy has shown benefits in pain reduction and daily functioning [42,127]. Rapid cycling hypobaric pressure may also relieve pain by decreasing inflammation, improving circulation, and enhancing tissue oxygenation, with alternating temperature shifts mimicking exercise-induced physiological effects [42].

When conservative treatments fail, surgical options such as excision or liposuction may be used to remove painful lipomas. These approaches can be effective but carry risks, including lipoma recurrence and post-surgical inflammation that may promote new lipoma formation [11,33,42]. While the precise mechanism of pain relief is unclear, disruption of nerve plexuses within fat tissue has been proposed, though the main benefit likely comes from reducing abnormal fat deposits, providing longer-lasting relief than temporary sensory loss [8].

Given the psychological impact of DD, mental health support is essential. Cognitive Behavioral Therapy for chronic pain improves functional outcomes even without significant pain reduction [128,129]. Psychosocial education, mindfulness, and management of central sensitization also contribute to symptom control [42,130,131]. A holistic approach addressing physical, emotional, and functional challenges is necessary for comprehensive care [8,15,132].

### 6.2. Molecular Targets and Novel Therapies

Emerging strategies for DD target the molecular pathways of inflammation and nociception. Anti-cytokine therapies, particularly those against TNF-α and IL-6, show promise. Infliximab, a TNF-α monoclonal antibody, reduces cytokine release, leukocyte migration, and endothelial permeability, alleviating neuropathic pain in other inflammatory conditions [13,133,134]. IL-6 receptor antagonists, such as tocilizumab and sarilumab, act on the Janus Kinase/Signal Transducer and Activator of Transcription (JAK-STAT) and MAPK pathways and represent potential treatments [14,135,136]. Methotrexate also exhibits immunomodulatory effects by inhibiting NF-κB, promoting T cell apoptosis, and dampening pro-inflammatory responses [14,137].

Parallel approaches target pain transmission pathways to address DD-associated hyperalgesia. Modulation of TRPV1 channels is under active investigation, with agonists such as capsaicin and resiniferatoxin (RTX) producing analgesia through nociceptor desensitization, while antagonists block calcium influx to reduce deep or visceral pain [88,138,139,140]. Inhibition of calcitonin gene-related peptide (CGRP) signaling has also shown anti-inflammatory effects and efficacy in migraine and disc herniation pain, suggesting potential applicability to DD [140,141,142,143,144].

Another promising direction involves modulating the immune microenvironment. Mast cells (MCs) and macrophages orchestrate an inflammatory response through mediators such as tryptase and Colony Stimulating Factor 1, influencing macrophage polarization and driving chronic inflammation, tissue remodeling, and immune dysregulation [145,146,147]. Interferon α-2b, by activating JAK-STAT signaling and reducing IL-1 and TNF production, further underscores the therapeutic relevance of cytokine modulation [13,14,42,148].

### 6.3. Metabolic Regulators and Lipid-Lowering Agents

Beyond anti-cytokine and nociceptive modulation, medications traditionally used for metabolic disorders may offer therapeutic benefits in Dercum’s disease by targeting adipogenesis, mitochondrial function, and systemic inflammation. Metformin, through activation of AMP-activated protein kinase (AMPK), promotes fatty acid oxidation, reduces macrophage polarization toward the pro-inflammatory M1 phenotype, and improves insulin sensitivity, collectively contributing to healthier adipose tissue remodeling [149,150]. GLP-1 receptor agonists such as liraglutide and semaglutide reduce body weight, enhance lipolysis, and attenuate circulating cytokines (IL-6 and TNF-α), which may indirectly mitigate pain and inflammation in DD [151,152].

In parallel, anti-hypercholesterolemic agents have potential relevance for preventing obesity-associated metabolic stress that may exacerbate lipomatosis. Statins exert pleiotropic anti-inflammatory actions beyond lipid lowering, including inhibition of NF-κB signaling and oxidative stress reduction in adipocytes [153]. PCSK9 inhibitors, by improving lipid profiles and reducing atherogenic lipoproteins, may attenuate lipotoxic stress within adipose tissue [154]. While direct clinical data in Dercum’s disease are lacking, these agents’ reproducible effects on adipose immunometabolism and systemic inflammation justify hypothesis-driven evaluation as adjunctive therapies.

### 6.4. Nutritional Modulation as a Complementary Therapy

Nutritional interventions are gaining increasing attention as complementary strategies for mitigating the inflammatory and metabolic disturbances characteristic of DD. Among these, the Mediterranean diet (MD) and plant-based dietary patterns have emerged as particularly promising due to their well-documented anti-inflammatory and metabolic effects. The MD emphasizes abundant intake of fruits, vegetables, legumes, nuts, whole grains, and olive oil, alongside moderate consumption of fish and poultry, and moderate wine consumption with meals. This dietary pattern provides a rich supply of monounsaturated and polyunsaturated fatty acids, dietary fiber, vitamins, and polyphenols [4,155,156,157]. Observational and interventional studies consistently link adherence to the MD with lower systemic inflammation, improved lipid profiles, and beneficial shifts in gut microbiota composition, which collectively confer protection against chronic diseases [158,159,160,161].

The anti-inflammatory effects of these dietary patterns are largely mediated by their bioactive components. Omega-3 fatty acids, sourced primarily from fish, nuts, and seeds, attenuate NF-κB activation, stimulate mitochondrial biogenesis, enhance fatty acid oxidation, and promote the synthesis of specialized pro-resolving mediators. This results in a measurable decrease in pro-inflammatory cytokines, including TNF-α, IL-6, IL-17, and IL-23 [162,163,164,165]. Similarly, polyphenolic compounds such as resveratrol, hydroxytyrosol, and quercetin modulate critical signaling pathways, including MAPK, JAK/STAT, and NF-κB, thereby reducing oxidative stress, restoring immune homeostasis, and enhancing regulatory T-cell activity [4,155]. Olive oil, a cornerstone of the MD, is particularly notable for its ability to improve cardiovascular health, optimize lipid metabolism, and promote microbial diversity in the gut, enriching beneficial taxa such as Lactobacillus, Bifidobacterium, and Bacteroides [166]. Dietary fiber further reinforces these benefits by fostering microbial diversity and driving the production of short-chain fatty acids, which enhance epithelial barrier integrity, upregulate anti-inflammatory cytokines such as IL-10, and contribute to systemic inflammation reduction [165,167,168,169]. Plant-based diets, including vegetarian and vegan patterns, have been associated with lower CRP levels, underscoring their anti-inflammatory potential [169,170,171,172,173].

Ketogenic dietary patterns have also demonstrated anti-inflammatory, metabolic, and therapeutic potential. These include the classical ketogenic diet (KD), modified Atkins diet (MAD), medium-chain triglyceride ketogenic diet, and very-low-carbohydrate ketogenic diet (VLCKD), and are characterized by carbohydrate consumption below 50 g per day or less than 10% of total caloric intake [155,174]. VLCKD, now termed very-low-energy ketogenic therapy, combines carbohydrate restriction with caloric control and has been shown to effectively reduce systemic inflammation, improve metabolic health, and support rapid and sustained weight reduction while preserving lean body mass and muscle performance [175]. Findings in other clinical contexts further demonstrate its anti-inflammatory effects in women with acne and an enhanced thyroid function in metabolic disorders [176,177]. At the mechanistic level, these diets promote a glucagon-dominant catabolic state, improve mitochondrial function, and decrease oxidative stress through β-hydroxybutyrate, which inhibits NF-κB signaling, NLRP3 inflammasome activation, and histone deacetylase activity [2]. Ketogenic regimens also modulate immune activity by enhancing regulatory T-cell responses and reducing pro-inflammatory cytokine production, contributing to broad anti-inflammatory effects [178,179]. Growing evidence has highlighted the role of inflammation in both the physiological and pathological mechanisms underlying chronic pain [91], and ketogenic dietary interventions have demonstrated potential in alleviating such pain, particularly in neurological disorders such as migraines and in the context of neurotraumatic injuries [180,181,182,183,184,185,186,187].

Other strategies to improve health and support weight management include caloric restriction (CR) and intermittent fasting (IF) [155]. CR involves reducing daily caloric in-take without causing malnutrition, typically by limiting overall food consumption, while IF achieves similar caloric reduction by skipping consecutive meals, making it more feasible for long-term adherence [188]. Both CR and IF effectively improve body weight, glycemic and lipid profiles, body composition, and blood pressure, with no significant differences between the two approaches [189,190]. The resulting fat mass loss contributes to lower adipokine levels and attenuated systemic inflammation, and IF has additionally demonstrated superior reductions in CRP in overweight individuals [155,188]. Another key factor is mitochondrial function, whose dysfunction in obesity contributes to chronic disease [191]. Sustained CR has been shown to reduce oxidative stress and mitochondrial DNA damage by 30% [192], while IF promotes efficient mitochondrial bioenergetics and modulates signaling pathways linked to metabolism, inflammation, and oxidative stress [191,193]. In immune cells, including monocytes, CR, IF, and KDs enhance mitochondrial performance [194,195].

Although evidence directly linking DD with specific dietary patterns is scarce [33,196], a case report noted pain exacerbation after sugary or fatty food intake [24], and dietary approaches emphasizing high plant sterols and low triglycerides have been suggested [197]. Research has also examined the MD, KD, and other low-carbohydrate regimens in related conditions such as lipedema [198,199,200,201,202,203,204].

## 7. Future Directions

The future of research and treatment in complex, multifactorial disorders must focus on building stronger foundations for data collection, molecular investigation, collaboration across specialties, and the development of targeted therapies. Progress to date has been slowed by fragmented approaches, inconsistent diagnostic practices, and a lack of standard frameworks for comparing outcomes across populations. To move forward, several priorities stand out as essential.

The first step is the establishment of large-scale patient registries supported by standardized diagnostic criteria [205,206]. At present, diagnosis often relies on exclusionary processes and subjective reports, which makes it difficult to determine who truly fits within a disease category [207]. This leads to wide heterogeneity across studies and pre-vents meaningful meta-analyses. Patient registries would solve multiple problems simultaneously, as they would provide structured systems for collecting demographic, clinical, and lifestyle information from diverse populations, while also allowing longitudinal follow-up that reveals how disease progresses and how patients respond to different treatments [208]. Registries would also streamline recruitment for clinical trials by making it easier to identify eligible participants who meet consistent criteria [209]. However, registries are only as good as the diagnostic frameworks that underpin them. Standardized diagnostic criteria must be developed collaboratively, involving clinicians, researchers, and patient advocacy groups [205]. These criteria should clearly define inclusion and exclusion thresholds, symptom scoring systems, and measures of disease severity. At the same time, they must remain flexible enough to incorporate emerging biomarkers as research advances. Harmonization across institutions and countries will ensure that data can be pooled and compared, accelerating discovery and translation into practice [210].

Alongside stronger clinical infrastructure, advances in molecular biology have opened new opportunities to study disease mechanisms in unprecedented depth [211]. Omics-based approaches, including transcriptomics, proteomics, and lipidomics, enable comprehensive profiling of affected tissues and can reveal insights invisible to conventional pathology [212,213]. For example, a study demonstrated that remodeling of the ECM in adipose and muscle tissue plays a pivotal role in metabolic disease development, identifying genes such as TCF7L2, ADIPOQ, CD36, PPARG, IL6, SIRT1, and COL5A1 as key regulators of inflammation [214]. Another investigation revealed that mitochondrial dysfunction in visceral adipose tissue not only disrupts lipid metabolism, but also exacerbates liver damage in metabolic dysfunction-associated steatohepatitis, highlighting the putative interconnectivity of adipose tissue and other organs [215]. These findings underscore the potential of integrative multiomic studies to uncover convergent pathways that may explain systemic symptoms, such as widespread pain and fatigue, in disorders like DD. Lipidomics adds another dimension by mapping lipid mediators that regulate inflammation and stress responses, potentially clarifying mechanisms behind symptom severity [216,217]. Collectively, these approaches can stratify patients into biologically defined subgroups, laying the foundation for precision medicine.

To note, advances in single-cell and spatial omics technologies have also uncovered extensive cellular heterogeneity in white adipose tissue, identifying over 60 subpopulations of adipocytes, stromal and adipogenic progenitors, and immune cells, with depot-specific differences that may influence metabolic and inflammatory states [218]. These insights could help identify progenitor cells with regenerative potential and reveal micro-environmental drivers of disease progression. As omics approaches become more refined, integrating transcriptomic, proteomic, and lipidomic analyses with spatial and single-cell data will be essential to capture dynamic cellular states and tissue-level interactions.

Progress in understanding and treating complex disorders will also require breaking down disciplinary silos [219]. Current care often fragments across specialties, leaving patients to cycle between rheumatologists, endocrinologists, pain specialists, and nutritionists without a unifying treatment plan [220]. A forward-looking model must integrate expertise from these different fields. Rheumatology brings insight into immune-mediated mechanisms and connective tissue biology, while endocrinology contributes knowledge of hormonal regulation, metabolic dysfunction, and stress axis abnormalities. Pain medicine provides tools for understanding nociceptive and neuropathic processes and designing multimodal pain management strategies. Nutrition science adds another essential dimension, linking dietary patterns and microbiome influences to metabolic and inflammatory states. Interdisciplinary collaboration benefits both clinical care and research [221]. Clinically, it makes it possible to design care plans that address multiple aspects of a patient’s condition simultaneously. For example, a patient with chronic pain, fatigue, and gastrointestinal complaints might receive a plan that integrates anti-inflammatory therapies, hormonal assessments, pain management, and dietary modification in a coordinated way. From a research perspective, interdisciplinary collaborations ensure that study designs capture the full complexity of the condition rather than reducing it to a single dimension [222]. Creating centers of excellence where specialists work together could provide hubs for both patient care and scientific innovation. These centers could also house biobanks, share standardized protocols, and offer training programs to cultivate the next generation of researchers skilled in interdisciplinary thinking.

Ultimately, the success of these efforts depends on translating mechanistic insights into treatments that improve lives, and this requires carefully designed clinical trials. Traditional pharmacological strategies have often failed to produce meaningful results in multifactorial systemic conditions, which underscores the need for innovation in trial de-sign. The following wo areas in particular deserve focus: diet-based therapies and molecularly targeted interventions. Diet-based therapies hold great promise because of their ability to modulate inflammation, metabolic pathways, and gut microbiome composition [223]. Trials could explore anti-inflammatory diets, elimination protocols, or macronutrient adjustments to see how these interventions affect symptoms and quality of life. The relative safety and long-term feasibility of dietary approaches make them attractive, but rigorous designs are necessary to account for adherence challenges, placebo effects, and variability across individuals. Molecularly targeted therapies, on the other hand, arise directly from omics-based discoveries. Small-molecule drugs, monoclonal antibodies, or biologic agents could be developed to modulate specific pathways implicated in the disease process. The key will be identifying biomarkers that can stratify patients, ensuring that targeted therapies are tested in the subgroups most likely to benefit. This precision approach increases the chances of success while minimizing unnecessary exposure to ineffective treatments [224,225].

Future clinical trials should also expand the range of outcomes measured. Symptom scores alone cannot capture the full impact of interventions. Trials should include molecular biomarkers, imaging data, functional capacity, and quality-of-life assessments. Adaptive trial designs may be especially useful, allowing researchers to adjust protocols as early results emerge, thus conserving resources while focusing on the most promising interventions. Patient advocacy groups and registry networks can play a pivotal role in trial design and recruitment, ensuring that studies address patient priorities and enroll diverse, representative populations [226,227].

Taken together, these strategies point toward a coherent vision for the future. Patient registries and standardized diagnostic criteria will generate reliable datasets for identifying subtypes and tracking outcomes. Omics technologies will provide the molecular detail needed to uncover mechanisms and therapeutic targets. Interdisciplinary collaboration will bring together the expertise necessary to manage complex, multifaceted conditions. Carefully designed clinical trials will translate discoveries into actionable treatments that can be tailored to individual patients. This vision is ambitious but achievable. It requires a commitment to collaboration, a willingness to embrace new technologies, and a patient-centered perspective that prioritizes meaningful outcomes. By aligning these efforts, the field can move decisively toward precision medicine and deliver tangible benefits to those affected.

## 8. Conclusions

DD exemplifies the complex interplay between adipose tissue dysfunction, chronic inflammation, and pain, positioning it as a distinctive model of painful, inflammatory obesity. Given its low prevalence, the literature on DD remains largely outdated, highlighting a pressing need for modern investigations into its pathophysiology. The current body of evidence suggests that adipokine imbalance, characterized by excess secretion of pro-inflammatory mediators, contributes to both local lipoma pathology and systemic low-grade inflammation. This inflammatory milieu appears to promote neuroimmune interactions, fueling neuroinflammation and the sensitization of nociceptors. Peripheral mechanisms, including altered activity of ion channels, further reinforce hyperexcitability within adipose tissue, while neuropeptides amplify pain perception. Together, these findings outline a mechanistic framework in which adipose tissue is not merely a passive site of fat accumulation, but an active driver of inflammation and nociceptive signaling.

Although progress has been made in identifying molecular patterns shared with other nociplastic and inflammatory conditions, major gaps remain in our understanding of the genetic and epigenetic contributions to DD. Reports of familial aggregation hint at heritable components, yet no definitive genetic markers have been established, and the potential role of obesity-induced epigenetic modifications remains unexplored. Similarly, therapeutic strategies continue to rely predominantly on symptomatic management, with limited success in addressing the underlying molecular drivers of the disease.

This landscape underscores the urgent need for robust mechanistic research and translational studies. Establishing patient registries, implementing omics-based analyses of affected tissues, and designing interdisciplinary clinical trials are essential steps toward clarifying disease biology and advancing treatment. By bridging the gap between clinical presentation and molecular mechanisms, future research can lay the foundation for precision-based strategies that move beyond symptomatic relief toward disease modification. Ultimately, unraveling the pathogenesis of DD may not only transform management for affected individuals, but also provide broader insights into the biology of obesity-associated pain and inflammation.

Nevertheless, several limitations should be acknowledged. The existing literature on Dercum’s disease is largely confined to case reports and small observational series, which restricts the strength and generalizability of current evidence. Mechanistic interpretations often rely on extrapolations from obesity and lipedema models, potentially overlooking disease-specific pathways. Diagnostic heterogeneity, lack of standardized outcome measures, and publication bias further complicate data comparison. Addressing these gaps through multicenter registries, molecular profiling, and controlled interventional studies will be critical to strengthen the evidence base and guide future therapeutic development.

## Figures and Tables

**Table 1:** Classification of Dercum disease.

	Subtype	Description
I	Generalized diffuse	Diffuse pain originating from fatty tissue, with tiny fat deposits that can be felt in different areas of the body, although discomfort may also be present in regions where no noticeable lumps are found [8,11,19].
II	Generalized nodular	Multiple localized areas throughout the body exhibit nodular formations, with pain occurring both within and surrounding the lipomas [8,11,19].
III	Localized nodular	Localized lipomas associated with pain in specific areas of the body [8,11,19].
IV	Juxta-articular	Painful fat folds situated within or adjacent to major joints such as the knee, hip, or elbow [8,11,19].

## Data Availability

No new data were created or analyzed in this study. Data sharing is not applicable to this article.

## Glossary

ASIC	Acid-Sensing Ion Channel
CAV1	Caveolin-1
COX	Cyclooxygenase
CR	Caloric Restriction
CRP	C-Reactive Protein
DD	Dercum’s Disease
ECM	Extracellular Matrix
ERα	Estrogen Receptor Alpha
ESR	Erythrocyte Sedimentation Rate
FML	Familial Multiple Lipomatosis
HIF1α	Hypoxia-Inducible Factor 1-Alpha
HMGA2	High Mobility Group AT-Hook 2
IF	Intermittent Fasting
IFN-γ	Interferon Gamma
IgG2c	Immunoglobulin G2c
IL	Interleukin
JAK-STAT	Janus Kinase/Signal Transducer and Activator of Transcription
KD	Ketogenic Diet
MAPK	Mitogen-Activated Protein Kinase
MD	Mediterranean Diet
MCP-1	Monocyte Chemoattractant Protein-1
NF-κB	Nuclear Factor Kappa-light-chain-enhancer of Activated B Cells
NGF	Nerve Growth Factor
NK	Natural Killer (cell)
NOS2	nitric oxide synthase 2
NSAID	Non-Steroidal Anti-Inflammatory Drug
PGE2	Prostaglandin E2
PKA	Protein Kinase A
SAT	Subcutaneous Adipose Tissue
SIRT1	Sirtuin 1
TGF-β	Transforming Growth Factor Beta
Th2	T Helper 2
TRPV1	Transient Receptor Potential Vanilloid 1
TNF-α	Tumor Necrosis Factor Alpha
VLCKD	Very Low-Carbohydrate Ketogenic Diet
